# An Advanced Backcross Population through Synthetic Octaploid Wheat as a “Bridge”: Development and QTL Detection for Seed Dormancy

**DOI:** 10.3389/fpls.2017.02123

**Published:** 2017-12-13

**Authors:** Zhang Dale, He Jie, Huang Luyu, Zhang Cancan, Zhou Yun, Su Yarui, Li Suoping

**Affiliations:** ^1^School of Life Science, Henan University, Kaifeng, China; ^2^Institute of Plant Stress Biology, Henan University, Kaifeng, China

**Keywords:** quantitative trait locus (QTL), *Aegilops tauschii*, pre-harvest sprouting, seed dormancy, synthetic octaploid wheat

## Abstract

The seed dormancy characteristic is regarded as one of the most critical factors for pre-harvest sprouting (PHS) resistance. As a wild wheat relative species, *Aegilops tauschii* is a potential genetic resource for improving common wheat. In this study, an advanced backcross population (201 strains) containing only *Ae. tauschii* segments was developed by means of synthetic octaploid wheat (hexaploid wheat Zhoumai 18 × *Ae. tauschii* T093). Subsequently, seed dormancy rate (Dor) in the advanced backcross population was evaluated on the day 3, 5 and 7, in which 2 major QTLs (*QDor-2D* and *QDor-3D*) were observed on chromosomes 2D and 3D with phenotypic variance explained values (PVEs) of 10.25 and 20.40%, respectively. Further investigation revealed significant correlation between *QDor-3D* and *Tamyb10* gene, while no association was found between the former and *TaVp1* gene, implying that *QDor-3D* site could be of closer position to *Tamyb10*. The obtained quantitative trait locus sites (QTLs) in this work could be applied to develop wheat cultivars with PHS resistance.

## Introduction

As a serious natural disaster, pre-harvest sprouting (PHS) is featured by seed germination occurring in spikes before wheat harvest (Sharma et al., 1994), leading to consumption of seed storage material, reduction of grain weight as well as degradation of nutrition and processing quality (Groos et al., 2002). In China, PHS has caused severe damage in many areas including the middle and lower reaches of the Yangtze river, southwest winter wheat and northeast spring wheat regions, due to conventional abundant rains in harvest season (Xiao et al., 2002). Unexpectedly, this situation has occasionally happened in the Huanghuai and north winter wheat regions in recent years. Therefore, breeding PHS resistant varieties has emerged as one of the major objectives to overcome this challenge, particularly for the wet and humid regions in harvest season (Liu et al., 2016).

Resistance to PHS is known to be linked to multi-factors including seed coat color, seed dormancy time, spike characteristics (spikelet density and awn length), germination inhibition substances of glume, alpha amylase activity, abscisic acid (ABA), and gibberellic acid (GA), etc., among which seed dormancy characteristic is regarded as one of the most critical factors for PHS resistance (Mares and Mrva, 2001; Gatford et al., 2002; Kottearachchi et al., 2006; Tan et al., 2006; Munkvold et al., 2009; Liu et al., 2017). Seed dormancy is a complex trait, as it can be affected by genetic background/gene combinations, as well as environmental conditions (Jaiswal et al., 2012; Kulwal et al., 2012). Therefore, a single major gene or quantitative trait locus (QTL) for seed dormancy cannot comprehensively explain the genetic diversity of wheat varieties. Up to now, QTLs for seed dormancy have been identified on each of the 21 chromosomes of wheat genome utilizing various mapping populations (Mares and Mrva, 2014). Most major QTLs are located on chromosomes 2B (Munkvold et al., 2009; Chao et al., 2010; Somyong et al., 2014), 3A (Mori et al., 2005; Liu et al., 2013), and 4A (Mares et al., 2005; Chen et al., 2008; Ogbonnaya et al., 2008; Torada et al., 2008; Mohan et al., 2009; Rasul et al., 2009; Cao et al., 2016). Major QTLs for PHS were also detected on chromosome 3D of red kernels wheat and 2D of synthetic hexaploid wheat (Groos et al., 2002; Ren et al., 2008). In addition, *TaVp1* and *Tamyb10* genes for PHS resistance have been identified in bread wheat, which are located on the long arms of chromosomes 3A, 3B, and 3D, respectively (Xia et al., 2009; Himi et al., 2011; Sun et al., 2012). *TaVp1* gene also performs the multi-functions of advancing embryo dormancy and repressing germination, besides promoting embryo maturation (McCarty et al., 1991). *Tamyb10* gene is found to be a transcription factor to regulate the flavonoid biosynthetic pathway, controlling proanthocyanidin synthesis in testa. PHS resistance is closely related with the red pigmentation, which could be possibly attributed to the pleiotropic effect of this gene (Himi et al., 2002).

*Aegilops tauschii* Cosson (DD, 2n = 2x = 14), the diploid progenitor of common wheat, is an annual, self-pollinated plant with high level of genetic variability for disease-resistance, productivity traits and abiotic stress resistance (Sukhwinder et al., 2012). It has a wide natural distribution in central Eurasia, spreading from northern Syria and Turkey to western China. In China, this species mainly distributes in Yili area of Xinjiang and middle reaches of the Yellow River (including Shanxi and Henan provinces) (Wei et al., 2008). The genetic variation of *Ae. tauschii* is more abundant than that of wheat D genome since only *Ae. tauschii* in certain distribution areas are involved in the origin of common wheat (Wang et al., 2013). Therefore, analogous to other wild crop progenitors, *Ae. tauschii* is considered as a prospective gene donor for improving common wheat (Kilian et al., 2011).

Many superior genes of *Ae. tauschii* have been transferred into common wheat by taking synthetic hexaploid wheat (tetraploid wheat × *Ae. tauschii*) as a “bridge” (Miranda et al., 2007). Actually, previous studies indicated that lots of QTLs from synthetic hexaploid wheat had been identified and some were found located on the D genome by utilizing advanced backcross population or introgression lines (Pestsova et al., 2006; Kunert et al., 2007; Naz et al., 2008; Yu et al., 2014). Alternatively, desirable traits may also be transferred from *Ae. tauschii* to common wheat via direct crossing (Miranda et al., 2007). Gill and Raupp (1987) proposed the first systematic direct gene transfer protocol. Though wheat genomes A, B, and D could be improved concurrently through hybridization of synthetic hexaploid wheat with common wheat, the interesting target alleles in *Ae. tauschii* could be transferred into common wheat through direct crossing, avoiding interference of adaptive allelic combinations from the other A and B genomes. However, only a few studies focused on this strategy due to its high sterility in hybrid F_1_ from distant hybridization and extremely low ripening rates in backcross between hybrid F_1_ and recurrent parent (Cox et al., 1990; Fritz et al., 1995; Olson et al., 2013). As a feature of this work, synthetic octaploid wheat (AABBDDDD, 2n = 8x = 56) was proposed as a “bridge” to overcome the above challenge through chromosome doubling of hybrid F_1_ obtained from the cross of *Ae. tauschii* and common wheat. In this study, an advanced backcross population containing only *Ae. tauschii* segments was developed through backcross of synthetic octaploid wheat with recurrent parent, which could effectively broaden the genetic background of common wheat. Meanwhile, QTLs for seed dormancy from *Ae. tauschii* were located in the population, and the obtained strains with seed dormancy characteristics could also provide novel genetic resource for PHS-resistance in wheat breeding.

## Materials and methods

### Plant material

**Figure 1:**
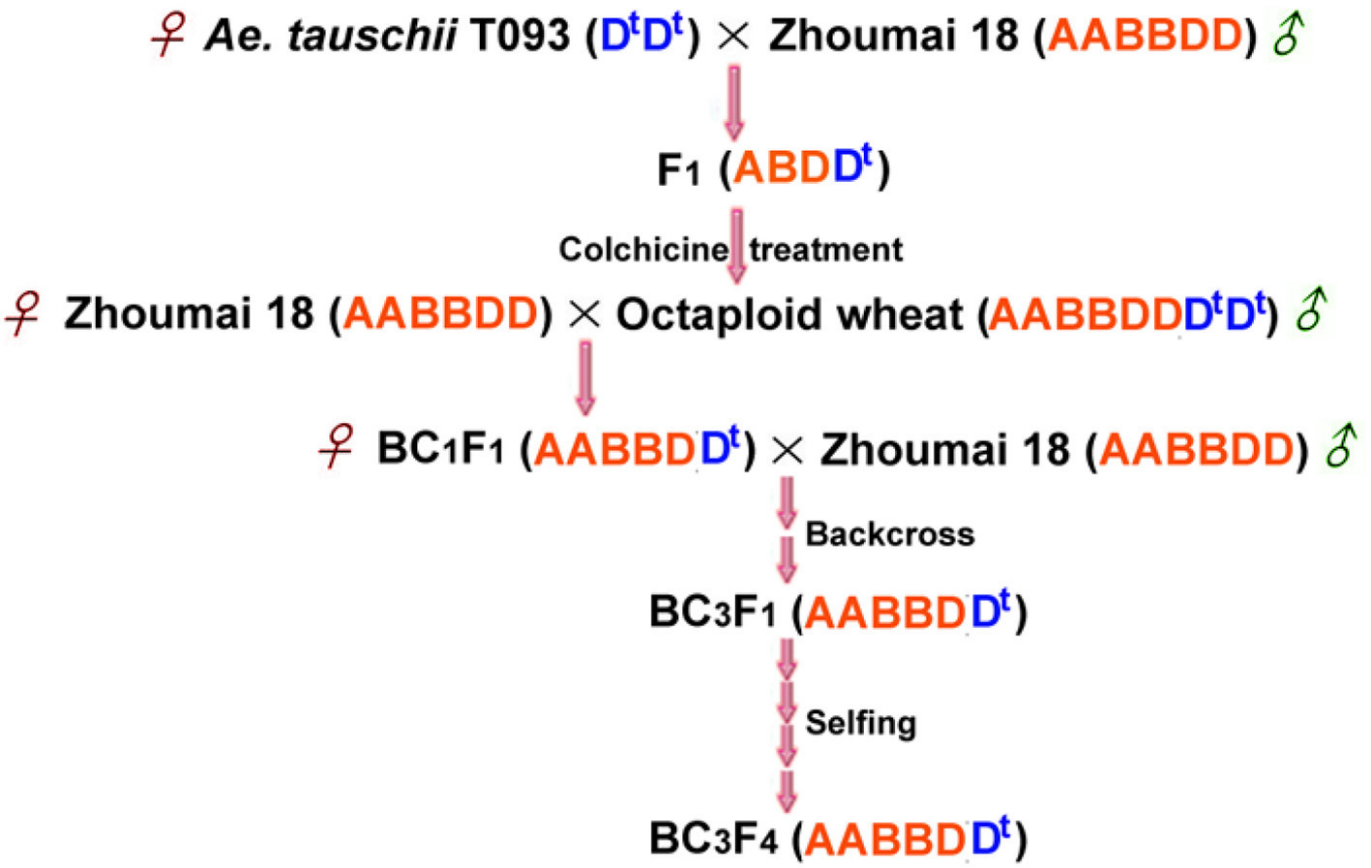
A crossing scheme for obtaining advanced backcross population through the “bridge” of synthetic octaploid wheat. D^t^ highlighted in blue designates the genome of *Ae. tauschii*.

The diploid *Ae. tauschii* ssp. *tauschii* accession T093 was originally derived from Henan province, which is resistant to PHS with long seed dormancy time after harvest. Zhoumai 18, a typical white-grain wheat with high susceptibility to PHS, was applied as recurrent parent in this work. Hybrid F_1_ plants were obtained through hybridization of *Ae. tauschii* accession T093 as female parents with Zhoumai 18, which were then treated with colchicine to generate synthetic octaploid wheat (AABBDDDD, 2n = 8x = 56). The next year, emasculated florets of Zhoumai 18 were pollinated by synthetic octaploid wheat to generate BC_1_F_1_ seeds. Afterwards, the BC_1_F_1_ plants, as female parents, were successively backcrossed two times by Zhoumai 18 and then selfed four generations to produce advanced backcross population (BC_3_F_4_ population) (Figure 1). Phenotypic traits of strains within the group were stabilized after several generations of backcross and selfing, demonstrating consistent ripening rates with the recurrent parent Zhoumai 18. The mapping population and Zhoumai 18 were cultivated on the 2014–2015 crop season in the wheat breeding farm of Plant Germplasm Resources and Genetic Engineering Laboratory, Henan University. Seeds were sown with 10 cm distance between plants and 30 cm row gap, which were grown under consistent field conditions.

### Map construction and QTL analysis

DNA was extracted from the fresh leaves of advanced backcross population and Zhoumai 18 in 2014 according to the method described previously (Olson et al., 2013). The genetic map was constructed based on the physical positions of simple sequence repeat (SSR) markers from wheat D genome (http://wheat.pw.usda.gov/cgi-bin/GG3/), in which the S19676-2 marker (F: CACTCAGCCAACCCAGGAAA, R: CAAATAGTTCTATCACTTGGTCTCCC) was exploited by utilizing the *Ae. tauschii* genome sequences (Jia et al., 2013). PCR reactions for SSR were performed using the method described by Röder et al. (1998). SSR markers were anchored and grouped to the seven *Ae. tauschii* chromosomes through sequence alignment between the primers and reference genome. The calculation of segment lengths and genome ratios was referred to the method described by Liu et al. (2006). The QTLs for seed dormancy were identified utilizing QTL IciMapping Ver 4.0 (Meng et al., 2015). RSTEP-LRT-ADD mapping (stepwise regression-based likelihood ratio test for additive QTL) was adopted and a significant threshold of likelihood of odds (LOD) was estimated by running 1,000 permutations with a type I error of 0.05.

### Amplification and analysis of *TaVp1* and *Tamyb10* genes

Two pairs of primers, *TaVp1-4-F1* (5′-TCTTGGTTCACTCGTTAGCATC-3′) + *Vp1-4-R1* (5′- CATTCTGCTCTTGTT GTTGGG-3′) and *Tamyb10-5-F1* (5′-AAGGAATGCGGCAAGAGTGA-3′) + *Tamyb10-5-R1* (5′-TCCTCCACGACCAAAGACCC-3′), were designed from the available sequences of *TaVp1D* (Genbank ID: AJ400714) and *Tamyb10-D1* (Genbank ID: KP279637), respectively. PCR reactions were performed using the method described by Röder et al. (1998). The physical positions of *TaVp1D* and *Tamyb10-D1* were determined based on the sequence alignment with *Ae. tauschii* reference genome. QTLs for seed dormancy of the former were checked in the advanced backcross population utilizing QTL IciMapping Ver 4.0. The correlation between PCR fragments from the latter and seed dormancy was analyzed by Wilcoxon rank sum test.

### Phenotypic evaluation

Five spikes from each line were harvested at day-40 post anthesis (40-dpa), and dried indoors for 5 days at ambient humidity and temperature, which were then manually threshed and placed at −20°C to preserve dormancy for 2 weeks due to slight differences in maturity. Fifty seeds were placed on moistened filter paper in a petri dish (150 mm diameter) and incubated in the dark. The Dor values were evaluated by germination test under room temperature (25°C) on the 3, 5, and 7 days, respectively, based on the method described by Cao et al. (2016) [Dor (%) = 100–GR (%) (GR: germination rate)]. The experiment was conducted with two replicates and the Dor was presented as the arithmetic mean values. The GR of each treatment was calculated using the following formula: GR = G/N, in which G and N stand for the numbers of germinated seeds and the total seeds in a given petri dish, respectively. With regard to lines with rather low germination rate value (≤5%), the remaining seeds were treated with 1 mL of 10 mM gibberellic acid and were then placed at 4°C for 3 days to break dormancy. Afterwards, they were transferred back at room temperature (25°C) and assessed for germination 10 days later. Lines which had not germinated were considered inviable and excluded for further calculation.

### Statistical analysis

Statistical analysis was performed on IBM® statistics 19 (SPSS Inc.,), including Friedman test, Wilcoxon rank sum test, and correlation coefficient (Pearson correlation). The significant difference of seed dormancy rate among correlated samples on the three detections was assessed by Friedman test, while that between two independent samples based on amplified fragments from *Tamyb10-5F1/Tamyb10-5R1* was evaluated by Wilcoxon rank sum test.

## Results

### Polymorphism marker on the D genome and number of introgressed segments

**Figure 2:**
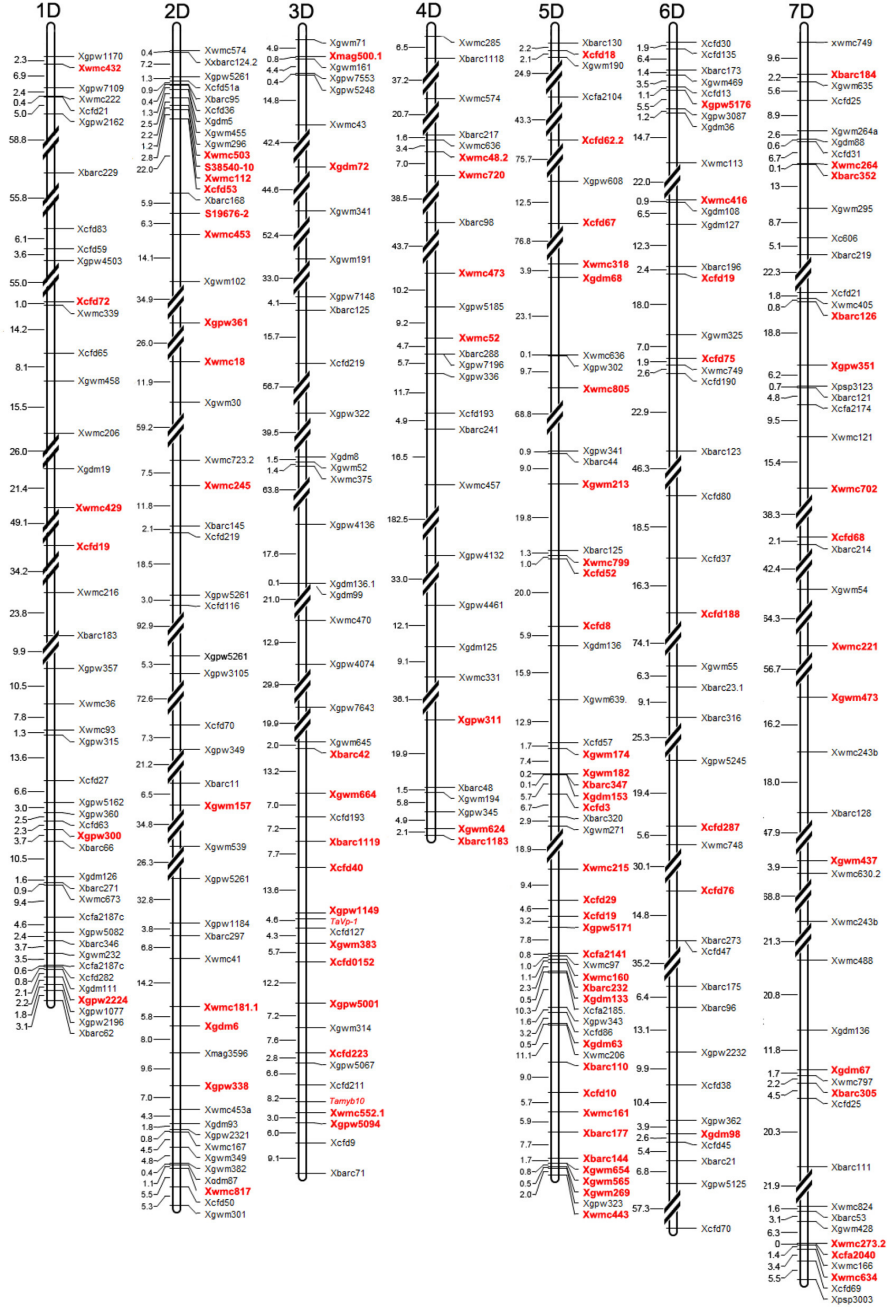
Physical map constructed based on the 304 polymorphic SSR markers between parents. Polymorphic markers in the advanced backcross population are highlighted in red. The unit of distance is megabasepairs (Mb).

Two hundred and one BC_3_F_4_ lines were successfully genotyped by SSR markers. Altogether 1114 SSR markers were used to detect polymorphism between the donor parent *Ae. tauschii* T093 and the recurrent parent Zhuomai 18. Among them, polymorphism between the two parents was detected in 374 SSR markers, in which 104 of them were confirmed to be polymorphic in the advanced backcross population, accounting for 27.8%, with an average of 14.9 markers for each chromosome (Table 1). Most of the polymorphic markers were observed on chromosome 5D with the total number of 36, whereas the least was found on chromosome 1D and 4D with the total number of only 6. Besides the unidentified 70 markers, a physical map was constructed based on the 304 polymorphic SSR markers between parents (Figure 2), which displayed heterogeneous distribution on 7 linkage groups of D genome, with a total length of 4004.5 Mb. The physical map illustrates an average interval of 36.2 Mb among 104 polymorphic markers in the population, while these markers also exhibit inhomogeneous distribution in different chromosome regions. Specifically, some markers concentrate in the same region with a very short distance, demonstrating a minimum gap of only 0.8 Mb or even no recombination events between them. However, huge long distances were also found for some other markers. For instance, the distance between markers *Xgdm72* and *Xbarc42* on chromosome 3D is determined to be 416.1 Mb.

**Table 1:** The size of introgressed segments detected in the advanced backcross population and cumulative proportion in the donor genome.

**Chr**.	**Polymorphic markers**	**Homozygous segments**		**Heterozygous segments**		**Maximum chromosome coverage (%)**
		**No. of segments**	**Average length (Mb)**	**No. of segments**	**Average length (Mb)**	
1D	6	23	27.5	12	21.9	31.2
2D	15	82	7.6	30	9.3	31.2
3D	16	212	11.3	33	8.1	32.5
4D	6	21	20.1	6	19.8	17.6
5D	36	686	8.4	27	7.3	75.6
6D	9	106	17.0	15	21.1	39.0
7D	16	233	16.1	36	6.7	44.3
Total	104	1363	15.4	159	13.5	37.0

Altogether 1,522 chromosome segments from *Ae. tauschii* were detected in the advanced backcross population (201 lines). Specifically, no segment was found in 39 lines (19.4%). While the remaining 162 lines (80.6%) contain 1363 homozygous and 159 heterozygous segments, with an average of 8.41 homozygous and 0.98 heterozygous segments in each line (Table S1, Figure S1). Only a single introgressed segment was observed in 34 lines, and 2 segments were identified in 19 lines. According to the physical positions of SSR markers, the size of each introgressed segment in the lines and ratios accounting for the whole donor genome were estimated (Table 1). The introgressed segments range from 1.0 to 60.5 Mb, with an average size of 15.4 Mb in homozygous and 13.5 Mb in heterozygous. In addition, the distribution of chromosome segment from *Ae. tauschii* exhibited a significant difference in wheat D genome (Figure S2). Typically, the introgression fragments from 4D of *Ae. tauschii* contain the least 27 fragments, only accounting for 1.8%. Whereas those from 5D occupy the most 713 fragments, accounting for 46.8%. The results herein clearly confirm that the chromosome segments of *Ae. tauschii* have been transferred into common wheat by means of synthetic octaploid wheat (*Ae. tauschii* T093 × Zhoumai18), which effectively broadens the genetic background of common wheat.

### Evaluation of seed dormancy rate

**Figure 3:**
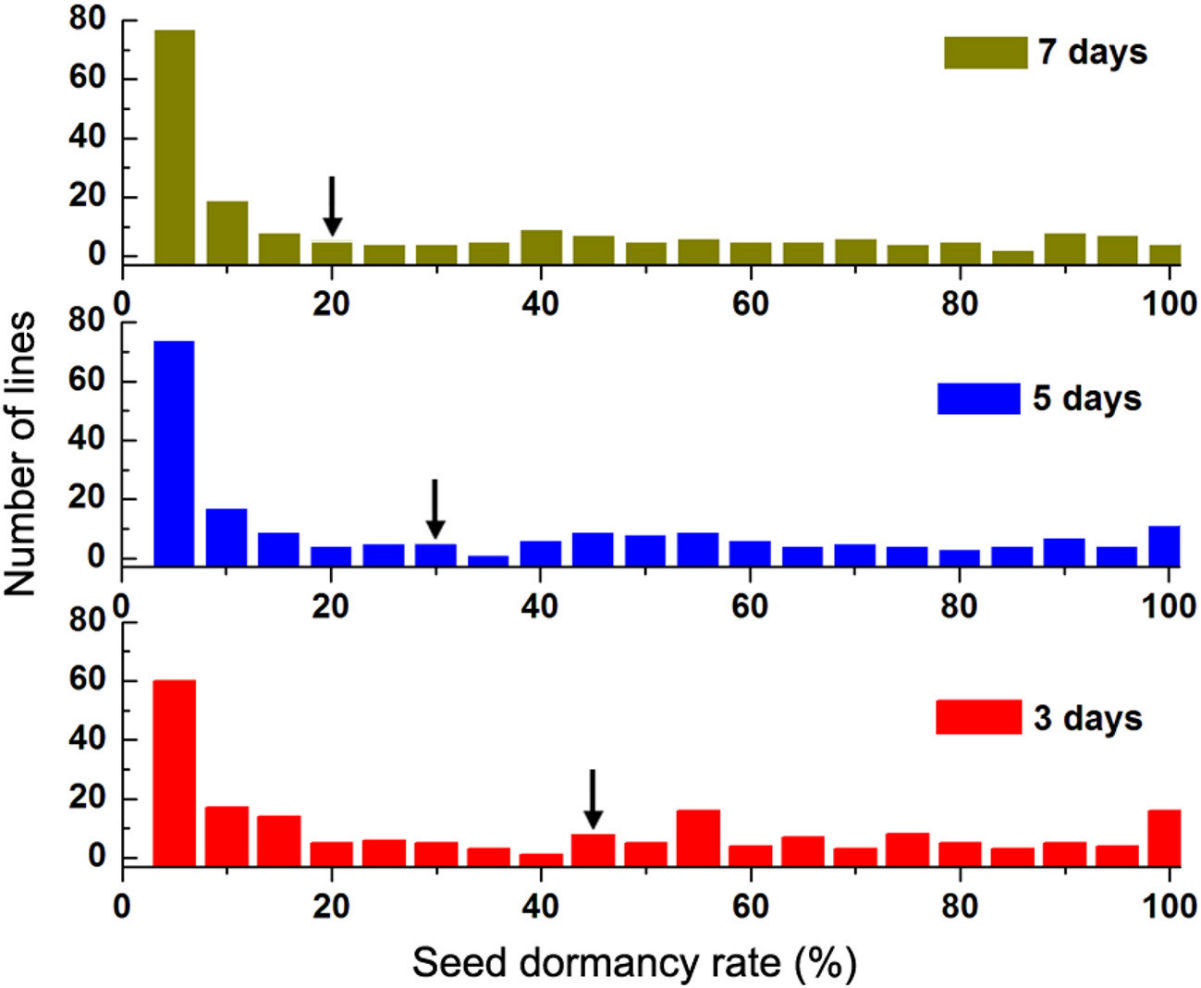
Frequency distributions of seed dormancy rate (Dor) of the advanced backcross population on the day 3, 5, and 7. Red: seed dormancy rate on the day 3; Blue: seed dormancy rate on the day 5; Green: seed dormancy rate on the day 7. Black arrows indicate the mean values of Dor from Zhoumai 18.

Seed dormancy rate (Dor) from 201 lines in the advanced backcross population was examined on the day 3, 5, and 7 (Table S2). Similar frequency distribution of Dor from 201 lines could be observed in the three detections (Figure 3), in which the most intensive distribution consistently located at rather low value (Dor ≤ 5%). For the long seed dormancy (Dor > 90%), the proportions were determined to be 10.9, 8.9, and 6.9%, respectively, for the day 3, 5, and 7. While as marked by the black arrows, the respective seed dormancy rates of the recurrent parent Zhoumai 18 were 44, 28, and 20% in the parallel experiment, indicating that the PHS-resistance strains are contained in the advanced backcross population. Phenotypic correlations among the obtained seed dormancy rates for the three detections were further analyzed through Pearson coefficient. As shown in Table 2, each pair of Dor exhibits high positive correlation with coefficient no <0.98. The correlation coefficients for 2 replicates were shown in Table S3. The higher positive correlation coefficient, the less Dor is affected by the external environment factors in this experiment. Additionally, Friedman test of Dor among the lines show highly significant difference (*P* < 0.01) for the three detections, implying minor phenotypic detection error for the obtained phenotype data.

**Table 2:** Correlation coefficients among three time periods associated with Dor in the advanced backcross population.

	**Dor 5**	**Dor 7**
Dor 3	0.989^**^	0.982^**^
Dor 5	-	0.996^**^

*Dor 3, seed dormancy rate on the day 3; Dor 5, seed dormancy rate on the day 5; Dor 7, seed dormancy rate on the day 7*;

** *,correlation is significant at the 0.01 level (2-tailed)*.

### QTL analysis of seed dormancy rate

**Figure 4:**
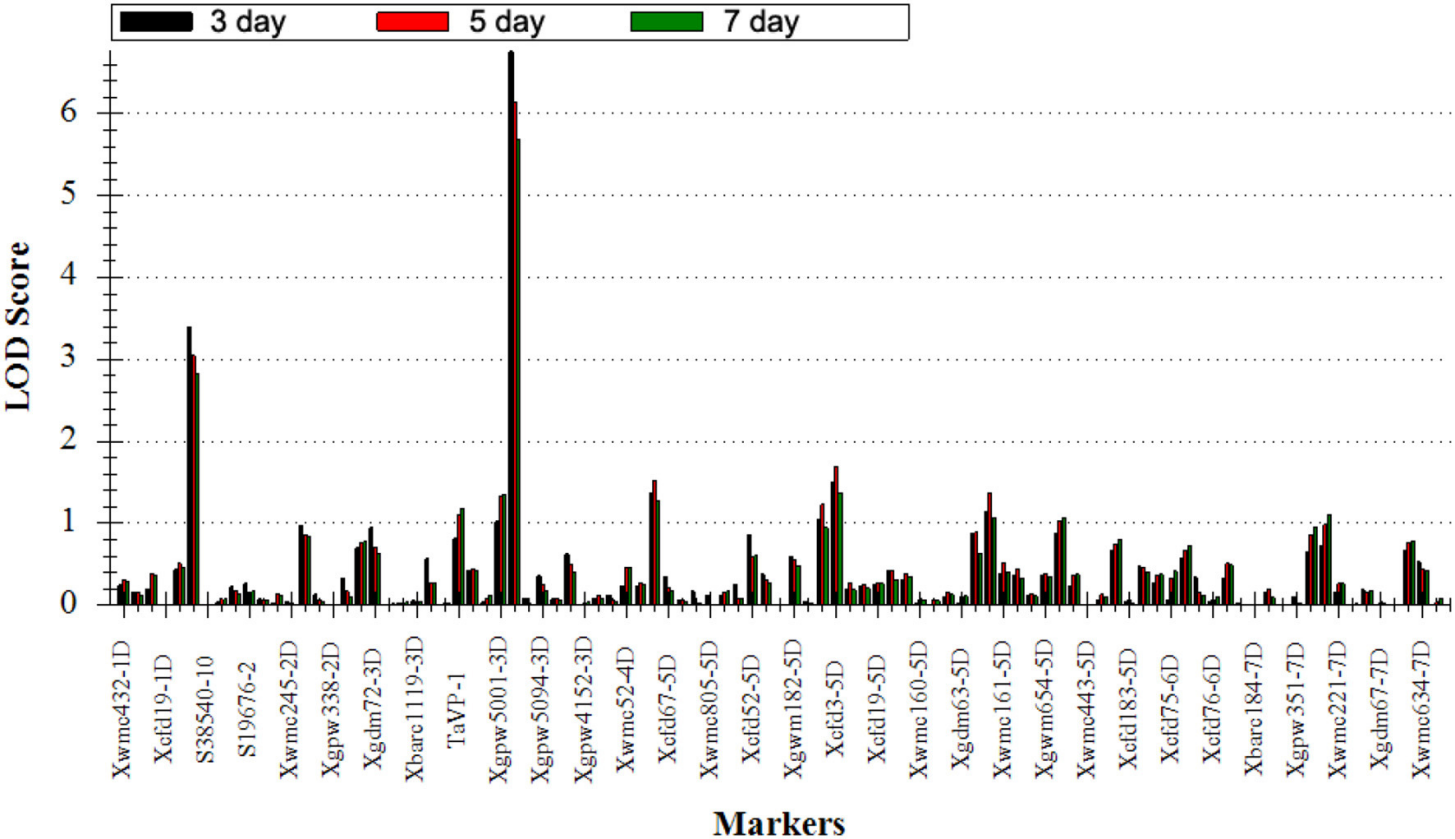
Positions of putative QTLs detected on the day 3, 5, and 7 in the advanced backcross population. Black: LOD of *QTLs* on the day 3; Red: LOD of *QTLs* on the day 5; Green: LOD of *QTLs* on the day 7.

To elucidate the genetic control for seed dormancy traits associated with PHS resistance, two major QTLs (*QDor-2D* and *QDor-3D*) were located on Xwmc503 of 2D and Xcfd223 of 3D by QTL IciMapping software in three detections under the single environment (Figure 4, Table 3). As listed in Table 3, the positive alleles of additive effect are derived from *Ae. tauschii*, further underscoring the valuable genes in *Ae. tauschii* as wheat wild resource (Sukhwinder et al., 2012). The *QDor-2D* displays the phenotypic variance explained values (PVEs) of 6.59, 6.02, and 5.64% in the three detections, respectively, corresponding to the additive effect values of 25.13, 23.50, and 22.02. As for *QDor-3D*, the PVE demonstrates prominently enhanced values of 13.83, 12.58, and 11.77%, with the additive effect values of 20.91, 19.50, and 18.27. Apparently, QTL detection for Dor could explain more phenotypic variance on the 3rd day compared with the other two measurements, implying the most prominent difference in the seed dormancy among lines in this detection.

**Table 3:** Analysis of putative QTLs for seed dormancy traits in the advanced backcross population.

**QTL**	**Detections**	**Position**	**Markers**	**LOD**	**PVE (%)**	**Add**
*QDor-2D*	3 day	2D (22.2 Mb)	Xwmc503	3.40	6.59	25.13
	5 day			3.04	6.02	23.50
	7 day			2.82	5.64	22.02
*QDor-3D*	3 day	3D (576.5 Mb)	Xcfd223	6.76	13.83	20.91
	5 day			6.13	12.58	19.50
	7 day			5.69	11.77	18.27
*TaVp-1*	3 day	3D (536.8 Mb)	Vp1-4	0.81	1.62	7.90
	5 day			1.10	2.20	9.00
	7 day			1.18	2.37	9.04

*LOD, likelihood of odds; PVE, phenotypic variance explained by each QTL; Add, additive effect. Positive values of Add indicate the effects increasing trait values by Ae. tauschii alleles, respectively*.

### Correlation analysis of *TaVp1, Tamyb10* genes and seed dormancy

**Figure 5:**
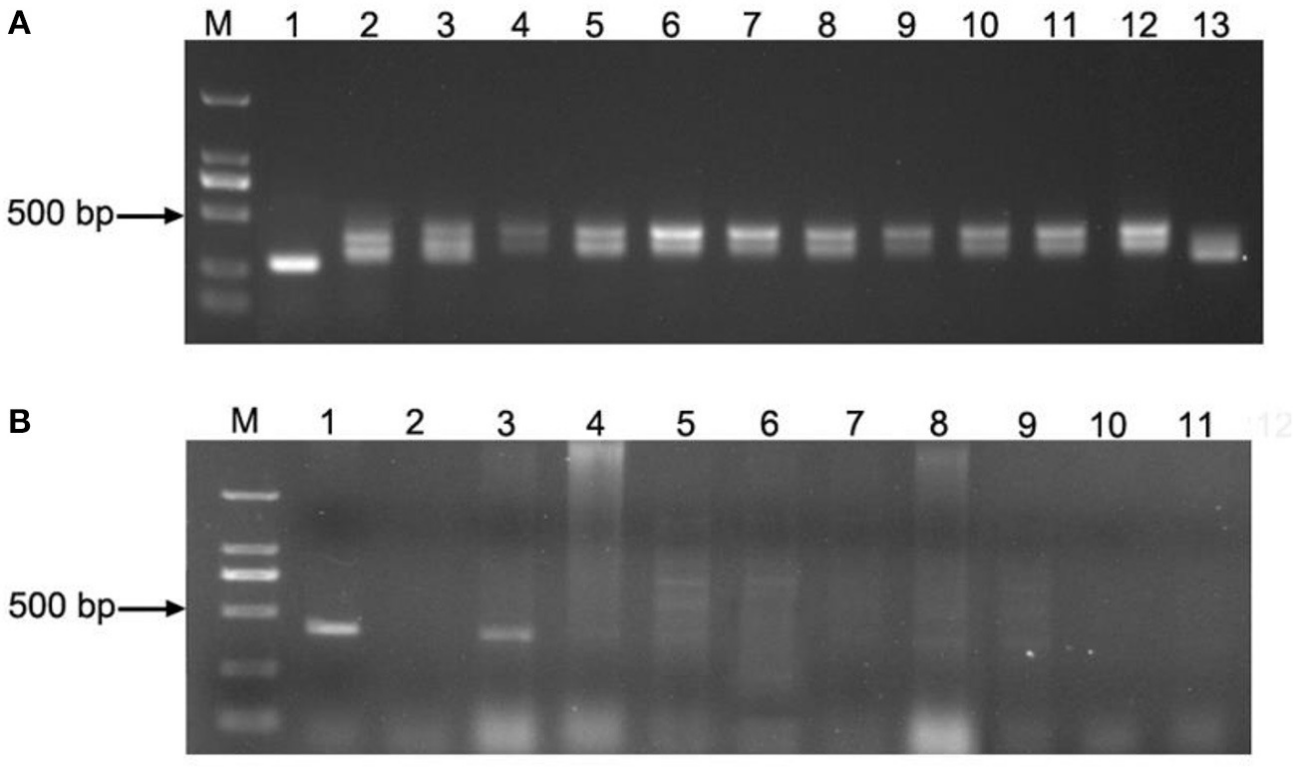
PCR amplification of *TaVp1* and *Tamyb10* sites in partial strains: *TaVp1* **(A)** (M: DL2000 marker; 1: *Ae. tauschii* (282 bp); 2: Zhoumai 18 (423 bp); 3–13: partial strains of advanced backcross population.); *Tamyb10* **(B)** (M: DL2000 marker; 1: *Ae. tauschii* (389 bp); 2: Zhoumai 18; 3–11: partial strains of advanced backcross population).

The genotypes of 201 strains in the advanced backcross population were analyzed through *TaVp1-4F1/TaVp1-4R1* and *Tamyb10-5F1/Tamyb10-5R1* primers (Figure 5). The former displayed co-dominant marker with two amplified fragments (282 bp from *Ae. tauschii* and 423 bp from Zhoumai 18), which was afterwards located on chromosome 3D by *Ae. tauschii* genome map (Figure 2). The LOD value of this site was found to be <3.0 (Table 3), demonstrating little correlation of *TaVp1* with seed dormancy traits. As could be observed in Figure 5, 389 bp fragment was amplified only from *Ae. tauschii* in the advanced backcross population by the dominant markers *Tamyb10-5F1/Tamyb10-5R1* since *Tamyb10-D1* gene in white-grained wheat varieties might be deleted. (Himi et al., 2011). Therefore, Wilcoxon rank sum test of two genotypes (0, 1) in the advanced backcross population was performed, revealing significant differences (*p* < 0.01) in seed dormancy between the two genotypes. This result implied that *Tamyb10-D1* gene may have prominent correlation with seed dormancy in the advanced backcross population.

## Discussion

Exploration and utilization of fine genes from *Ae. tauschii* is an effective approach to improve the resistance of common wheat, especially in view of the drastic reduction in genetic diversity due to modern breeding (Sukhwinder et al., 2012). Meanwhile, it is convenient to transfer *Ae. tauschii* genes into common wheat by recombination between homologous chromosomes, and most possibly, undesirable gene linkages could be easily broken by repeated backcross with common wheat. Direct crossing from diploid species into hexaploid wheat has been applied as a possible plant breeding technique for rapid introgression of useful traits. Gill and Raupp (1987) provided the first systematic direct gene transfer protocol. Based on this perspective, BC_2_F_1_ population was constructed through direct crossing of Ug99-resistant *Ae. tauschii* with rust-susceptible wheat (Olson et al., 2013). Another work of direct crossing was reported by Sehgal et al. (2011), who constructed BC_1_F_4_ population derived from the cross of three heat-tolerant *Ae. tauschii* with bread wheat. In this work, advanced backcross population of BC_3_F_4_ was constructed through synthetic octaploid wheat as a “bridge,” which was obtained from chromosome doubling of hybrid F_1_ through direct crossing of *Ae. tauschii* T093 with common wheat Zhoumai 18. Meanwhile, many strains with poor comprehensive traits could be eliminated in the process of multigenerational backcross and selfing. Therefore, only 27.8% SSR markers were detected in the advanced backcross population, though 374 SSR markers between *Ae. tauschii* accession T093 and Zhoumai 18 were determined to be polymorphic. While in another aspect, the reserved strains may possess better agronomic traits, and no phenotype segregation was found in each line, indicating that these lines are cytogenetically stable, which could be utilized more easily through further breeding.

Seed dormancy is widely regarded as one of the most critical factors for PHS resistance in common wheat, which is greatly influenced by temperature in seed germination stage. Specially, high temperature (>26°C) has negative influence on seed dormancy in late development stage (Ueno, 2002). In this work, the experiment was conducted at ~25°C, very close to that proposed in the previous literature (Ueno, 2002). The strains with high Dor values could still be detected in this case, revealing the strong additive effect of QTL for seed dormancy in the advanced backcross population. These strains containing desirable seed dormancy characterization herein could provide valuable genes for PHS-resistance breeding.

It is well known that wheat grain color (GC) is linked to PHS-resistance, and red-grained wheat is of more PHS resistance than the white-grained one (Flintham, 2000; Warner et al., 2000; Himi et al., 2002). Early cytogenetic study suggested that GC was controlled by three genes, *R-A1, R-B1*, and *R-D1*, locating on homoeologous group 3 chromosomes (Metzger and Silbaugh, 1970). Groos et al. (2002) detected 3 QTLs for PHS locating on the long arm of chromosomes 3A, 3B, and 3D in a bi-parental population, closing to the loci of genes *R* and *TaVp1*, with boundary from *Xgwm314* to *Xcfd9* on chromosome 3D and marker interval of 37.9 cM. The additive effect of QTLs was attributed to the wheat variety “‘Renan” with red kernels. Lin et al. (2016) also found 3 QTLs for GC on chromosome 3A, 3B, and 3D in 185 wheat cultivars by genome-wide association study (GWAS). Among the 3 sites, *Tamyb10-D1* demonstrated the highest effect on GC (*R*^2^ = 0.23) in the association mapping panel. Moreover, the corresponding *Qphs.hwwgr-3DL* for PHS-resistance was also observed on chromosome 3D, explaining PVE of 8.3%, which suggests the pleiotropic effects of *Tamyb10-D1* on PHS resistance under the field conditions. In this study, the additive effect of *QDor-3D* origins from *Ae. tauschii*, with the highest value of 20.91 and PVE of 13.83% (higher than the PVE of *Qphs.hwwgr-3DL*). Further analysis indicates that *Tamyb10-D1* from *Ae. tauschii* is highly related with seed dormancy (*p* < 0.01), while no correlation was found between *TaVp1* and seed dormancy. A novel candidate gene is thus speculated to exist between *QDor-3D* (576.5 Mb) and *Tamyb10-D1* (583.9 Mb) for rather large distance (7.4 Mb) in physical position and the little difference in testa color (yellow) among the lines.

QTLs for PHS on chromosome 2D have been identified in recent years. Through a DH population from cross of wheat Cascades with AUS1408, Tan et al. (2006) found a major QTL locating on chromosome 2D (marker interval *Xwmc112*-*Xgwm102*), with enhanced dormancy from the allele of Cascades. Ren et al. (2008) detected a major QTL (*Qphs.sau-2D*) for PHS-resistance on the short arm of chromosome 2D in 140 F_2_ plants. The *Qphs.sau-2D* was identified within the marker interval of *Xwmc261*-*Xgwm484*, with genetic distance of 15.4 cM, whose additive effect was established to be derived from the D genome of synthetic hexaploid wheat cultivar “RSP.” In this study, the additive effect of *QDor-2D* originates from *Ae. tauschii*, with the highest value of 25.19 and PVE of 20.40 %, which was located on Xwmc503 of 2D, which was completely included in Xgwm261-Xgwm484 based on the *Ae. tauschii* reference genome. Specifically, the physical positions of Xwmc503 and Xgwm261 are respectively located in 22226966 ~ 22227188 bp and 22218627 ~ 22218480 bp, with a strikingly close distance of 8 Kb. QTLs for PHS-resistance could be found at analogous positions on 2D by utilizing different mapping populations, providing a strong evidence of the existence of candidate genes for PHS-resistance nearby. From this point of view, the *QDor-2D* identified in this study is suitable for marker-assisted breeding to trace the *Ae. tauschii* segment with seed dormancy characterization.

In conclusion, an advanced backcross population containing only *Ae. tauschii* segments was established through the synthetic octaploid wheat (hexaploid wheat Zhoumai 18 × *Ae. tauschii* T093) as a “bridge.” Meanwhile, 2 major QTLs (*QDor-2D* and *QDor-3D*) for seed dormancy from *Ae. tauschii* were located on Xwmc503 of 2D and Xcfd223 of 3D through the advanced backcross population, respectively. These QTLs could provide valuable information for marker-assisted breeding, and the obtained strains with long seed dormancy may also provide novel genetic resource for PHS-resistance in wheat breeding.

## Author contributions

Conceived and designed the study: LS. Generated the data and performed the analysis: ZD, HJ, HL, ZC, and LS. Contributed reagents, materials, analysis tools: ZY and SY. Wrote the paper: ZD and LS. All authors read and approved the final manuscript.

### Conflict of interest statement

The authors declare that the research was conducted in the absence of any commercial or financial relationships that could be construed as a potential conflict of interest.

## References

[B1] Cao L. Hayashi K. Tokui M. Mori M. Miura H. Onishi K. (2016). Detection of QTLs for traits associated with pre-harvest sprouting resistance in bread wheat (*Triticum aestivum* L.). Breed. Sci. 66, 260–270. 10.1270/jsbbs.66.260 27162497 PMC4785003

[B2] Chao S. Xu S. S. Elias E. M. Faris J. D. Sorrells M. E. (2010). Identification of chromosome locations of genes affecting preharvest sprouting and seed dormancy using chromosome substitution lines in tetraploid wheat (*Triticum turgidum* L.). Crop Sci. 50, 1180–1187. 10.2135/cropsci2009.10.0589

[B3] Chen C. X. Cai S. B. Bai G. H. (2008). A major QTL controlling seed dormancy and pre-harvest sprouting resistance on chromosome 4A in a Chinese wheat landrace. Mol. Breed. 21, 351–358. 10.1007/s11032-007-9135-5

[B4] Cox T. S. Hatcher J. H. Gill B. S. Raupp W. J. Sears R. G. (1990). Agronomic performance of hexaploid wheat lines derived from direct crosses between wheat and *Aegilops squarrosa*. Plant Breed. 105, 271–277. 10.1111/j.1439-0523.1990.tb01285.x

[B5] Flintham J. E. (2000). Different genetic components control coat-imposed and embryo-imposed dormancy in wheat. Seed Sci. Res. 10, 43–50. 10.1017/S0960258500000052

[B6] Fritz A. K. Cox T. S. Gill B. S. Sears R. G. (1995). Molecular marker-facilitated analysis of introgression in winter wheat × *Triticum tauschii* populations. Crop Sci. 35, 1691–1695. 10.2135/cropsci1995.0011183X003500060030x

[B7] Gatford K. T. Eastwood R. F. Halloran G. M. (2002). Germination inhibitors in bracts surrounding the grain of *Triticum tauschii*. Funct. Plant Biol. 29, 881–890. 10.1071/PP01011 32689536

[B8] Gill B. S. Raupp W. J. (1987). Direct genetic transfers from *Aegilops squarrosa* L. to hexaploid wheat. Crop Sci. 27, 445–450. 10.2135/cropsci1987.0011183X002700030004x

[B9] Groos C. Gay G. Perretant M. R. Bernard G. M. Dedryver F. Charmet G. (2002). Study of the relationship between pre-harvest sprouting and grain color by quantitative trait loci analysis in a white × red grain bread-wheat cross. Theor. Appl. Genet. 104, 39–47. 10.1007/s001220200004 12579426

[B10] Himi E. Maekawa M. Miura H. Noda K. (2011). Development of PCR markers for *Tamyb10* related to R-1, red grain color gene in wheat. Theor. Appl. Genet. 122, 1561–1576. 10.1007/s00122-011-1555-2 21359957

[B11] Himi E. Mares D. J. Yanagisawa A. Noda K. (2002). Effect of grain colour gene (R) on grain dormancy and sensitivity of the embryo to abscisic acid (ABA) in wheat. J. Exp. Bot. 53, 1569–1574. 10.1093/jxb/erf005 12096095

[B12] Jaiswal V. Mir R. R. Mohan A. Balyan H. S. Gupta P. K. (2012). Association mapping for pre-harvest sprouting tolerance in common wheat (*Triticum aestivum* L.). Euphytica 188, 89–102. 10.1007/s10681-012-0713-1

[B13] Jia J. Z. Zhao S. C. Kong X. Y. Li Y. R. Zhao G. Y. He W. M.. (2013). *Aegilops tauschii* draft genome sequence reveals a gene repertoire for wheat adaptation. Nature 496, 91–95. 10.1038/nature12028 23535592

[B14] Kilian B. Mammen K. Millet E. Sharma R. Graner A. Salamini F. (2011). Aegilops, in Wild Crop Relatives: Genomic and Breeding Resources Cereals, ed Kole C. (Berlin: Springer), 1–76.

[B15] Kottearachchi N. S. Uchino N. Kato K. Miura H. (2006). Increased grain dormancy in white-grained wheat by introgression of pre-harvest sprouting tolerance QTLs. Euphytica 152, 421–428. 10.1007/s10681-006-9231-3

[B16] Kulwal P. Ishikawa G. Benscher D. Feng Z. Yu L. X. Jadhav A.. (2012). Association mapping for pre-harvest sprouting resistance in white winter wheat. Theor. Appl. Genet. 125, 793–805. 10.1007/s00122-012-1872-0 22547141

[B17] Kunert A. Naz A. A. Dedeck O. Pillen K. Léon J. (2007). AB-QTL analysis in winter wheat: I. Synthetic hexaploid wheat (*T. turgidum* ssp. *dicoccoides* × *T. tauschii*) as a source of favourable alleles for milling and baking quality traits. Theor. Appl. Genet. 115, 683–695. 10.1007/s00122-007-0600-7 17634917

[B18] Lin M. Zhang D. D. Liu S. B. Zhang G. R. Yu J. M. Fritz A. K.. (2016). Genome-wide association analysis on pre-harvest sprouting resistance and grain color in U.S. winter wheat. BMC Genomics 17:794. 10.1186/s12864-016-3148-6 27729004 PMC5059910

[B19] Liu C. X. Ding F. Hao F. H. Yu M. Lei H. H. Wu X. Y.. (2016). Reprogramming of seed metabolism facilitates pre-harvest sprouting resistance of wheat. Sci. Rep. 6:20593. 10.1038/srep20593 26860057 PMC4748292

[B20] Liu S. B. Sehgal S. K. Li J. R. Lin M. Trick H. N. Yu J. M. (2013). Cloning and characterization of a critical regulator for pre-harvest sprouting in wheat. Genetics 195, 263–273. 10.1534/genetics.113.152330 23821595 PMC3761307

[B21] Liu S. Zhou R. Dong Y. Li P. Jia J. (2006). Development, utilization of introgression lines using a synthetic wheat as donor. Theor. Appl. Genet. 112, 1360–1373. 10.1007/s00122-006-0238-x 16550399

[B22] Liu Y. Liu Y. Zhou Y. Wight C. Pu Z. Qi P. (2017). Conferring resistance to pre-harvest sprouting in durum wheat by a QTL identified in *Triticum spelta*. Euphytica 213, 19 10.1007/s10681-016-1796-x

[B23] Mares D. J. Mrva K. (2001). Mapping quantitative trait loci associated with variation in grain dormancy in Australian wheat. Aust. J. Agric. Res. 52, 1257–1265. 10.1071/AR01049

[B24] Mares D. J. Mrva K. (2014). Wheat grain pre-harvest sprouting and late maturity alpha-amylase. Planta 240, 1167–1178. 10.1007/s00425-014-2172-5 25257145

[B25] Mares D. J. Mrva K. Cheong J. Williams K. Watson B. Storlie E. (2005). A QTL located on chromosome 4A associated with dormancy in white and red grained wheat of diverse origin. Theor. Appl. Genet. 111, 1357–1364. 10.1007/s00122-005-0065-5 16133305

[B26] McCarty D. R. Hattori T. Carson C. B. Vasil V. Lazar M. Vasil I. K. (1991). The viviparous-1 developmental gene of maize encodes a novel transcriptional activator. Cell 66, 895–905. 10.1016/0092-8674(91)90436-3 1889090

[B27] Meng L. Li H. H. Zhang L. Y. Wang J. K. (2015). QTL IciMapping: Integrated software for genetic linkage map construction and quantitative trait locus mapping in biparental populations. Crop J. 3, 269–283. 10.1016/j.cj.2015.01.001

[B28] Metzger R. J. Silbaugh B. A. (1970). Location of genes for seed coat color in hexaploid wheat, *Triticum aestivum* L. Crop Sci. 10, 495–496. 10.2135/cropsci1970.0011183X001000050012x

[B29] Miranda L. M. Murphy J. P. Marshall D. Cowger C. Leath S. (2007). Chromosomal location of Pm35, a novel *Aegilops tauschii* derived powdery mildew resistance gene introgressed into common wheat (*Triticum aestivum* L.). Theor. Appl. Genet. 114, 1451–1456. 10.1007/s00122-007-0530-4 17356863

[B30] Mohan A. Kulwal P. Singh R. Kumar V. Mir R. R. Kumar J. (2009). Genome-wide QTL analysis for pre-harvest sprouting tolerance in bread wheat. Euphytica 168, 319–329. 10.1007/s10681-009-9935-2

[B31] Mori M. Uchino N. Chono M. Kato K. Miura H. (2005). Mapping QTLs for grain dormancy on wheat chromosome 3A and group 4 chromosomes, and their combined effect. Theor. Appl. Genet. 110, 1315–1323. 10.1007/s00122-005-1972-1 15803290

[B32] Munkvold J. D. Tanaka J. Benscher D. Sorrells M. E. (2009). Mapping quantitative trait loci for preharvest sprouting resistance in white wheat. Theor. Appl. Genet. 119, 1223–1235. 10.1007/s00122-009-1123-1 19669633

[B33] Naz A. A. Kunert A. Lind V. Pillen K. Léon J. (2008). AB-QTL analysis in winter wheat: II. Genetic analysis of seedling and field resistance against leaf rust in a wheat advanced backcross population. Theor. Appl. Genet. 116, 1095–1104. 10.1007/s00122-008-0738-y 18338154 PMC2358941

[B34] Ogbonnaya F. C. Imtiaz M. Ye G. Hearnden P. R. Hernandez E. Eastwood R. F.. (2008). Genetic and QTL analyses of seed dormancy and preharvest sprouting resistance in the wheat germplasm CN10955. Theor. Appl. Genet. 116, 891–902. 10.1007/s00122-008-0712-8 18368385

[B35] Olson E. L. Rouse M. N. Pumphrey M. O. Bowden R. L. Gill B. S. Poland J. A. (2013). Simultaneous transfer, introgression, and genomic localization of genes for resistance to stem rust race TTKSK (Ug99) from *Aegilops tauschii* to wheat. Theor. Appl. Genet. 126, 1179–1188. 10.1007/s00122-013-2045-5 23377571

[B36] Pestsova E. G. Börner A. Röder M. S. (2006). Development and QTL assessment of *Triticum aestivum*-*Aegilops tauschii* introgression lines. Theor. Appl. Genet. 112, 634–647. 10.1007/s00122-005-0166-1 16341683

[B37] Rasul G. Humphreys D. G. Brûlé-Babel A. McCartney C. A. Knox R. E. DePauw R. M. (2009). Mapping QTLs for pre-harvest sprouting traits in the spring wheat cross “RL4452/AC Domain”. Euphytica 168, 363–378. 10.1007/s10681-009-9934-3

[B38] Ren X. B. Lan X. J. Liu D. C. Wang J. L. Zheng Y. L. (2008). Mapping QTLs for pre-harvest sprouting tolerance on chromosome 2D in a synthetic hexaploid wheat × common wheat cross. J. Appl. Genet. 49, 333–341. 10.1007/BF03195631 19029680

[B39] Röder M. S. Korzun V. Wendehake K. Plaschke J. Tixier M. H. Leroy P.. (1998). A microsatellite map of wheat. Genetics 149, 2007–2023. 9691054 10.1093/genetics/149.4.2007 PMC1460256

[B40] Sehgal S. K. Kaur S. Gupta S. Sharma A. Kaur R. Bains N. S. (2011). A direct hybridization approach to gene transfer from *Aegilops tauschii* Coss. to *Triticum aestivum* L. Plant Breed. 130, 98–100. 10.1111/j.1439-0523.2010.01817.x

[B41] Sharma S. K. Dhaliwal H. S. Multani D. S. Bains S. S. (1994). Inheritance of pre-harvest sprouting tolerance in *Triticum aestivum* and its transfer to an amber-grained cultivar. J. Hered. 85, 312–314. 10.1093/oxfordjournals.jhered.a111466

[B42] Sukhwinder S. Chahal G. S. Singh P. K. Gill B. S. (2012). Discovery of desirable genes in the germplasm pool of *Aegilops tauschii* Coss. Indian J Genet. 72, 271–277.

[B43] Somyong S. Ishikawa G. Munkvold J. D. Tanaka J. Benscher D. Cho Y. G.. (2014). Fine mapping of a preharvest sprouting QTL interval on chromosome 2B in white wheat. Theor. Appl. Genet. 127, 1843–1855. 10.1007/s00122-014-2345-4 24985065

[B44] Sun Y. W. Yang Y. Shewry P. R. Jones H. D. Xia L. Q. (2012). Isolation and characterization of *Viviparous-1* haplotypes in wheat related species. Euphytica 188, 71–84. 10.1007/s10681-012-0659-3

[B45] Tan M. K. Sharp P. J. Lu M. Q. Howes N. (2006). Genetics of grain dormancy in a white wheat. Aust. J. Agri. Res. 57, 1157–1165. 10.1071/AR06101

[B46] Torada A. Koike M. Ikeguchi S. Tsutsui I. (2008). Mapping of a major locus controlling seed dormancy using backcrossed progenies in wheat (*Triticum aestivum* L.). Genome 51, 426–432. 10.1139/G08-007 18521121

[B47] Ueno K. (2002). Effects of desiccation and a change in temperature on germination of immature grains of wheat (*Triticum aestivum* L.). Euphytica 126, 107–113. 10.1023/A:1019655218722

[B48] Wang J. R. Luo M. C. Chen Z. X. You F. M. Wei Y. M. Zheng Y. L.. (2013). *Aegilops tauschii* single nucleotide polymorphisms shed light on the origins of wheat D-genome genetic diversity and pinpoint the geographic origin of hexaploid wheat. New Phytol. 198, 925–937. 10.1111/nph.12164 23374069

[B49] Warner R. L. Kudrna D. A. Spaeth S. C. Jones S. S. (2000). Dormancy in white-grain mutants of Chinese Spring wheat (*Triticum aestivum* L.). Seed Sci. Res. 10, 51–60. 10.1017/S0960258500000064

[B50] Wei H. T. Li J. Peng Z. S. Lu B. R. Zhao Z. J. Yang W. Y. (2008). Relationships of *Aegilops tauschii* revealed by DNA fingerprints: The evidence for agriculture exchange between China and the West. Prog. Nat. Sci. 18, 1525–1531. 10.1016/j.pnsc.2008.05.022

[B51] Xia L. Q. Yang Y. Ma Y. Z. Chen X. M. He Z. H. Röder M. S. (2009). What can the *Viviparous-1* gene tell us about wheat pre-harvest sprouting? Euphytica 168, 385–394. 10.1007/s10681-009-9928-1

[B52] Xiao S. H. Zhang X. Y. Yan C. S. Lin H. (2002). Germplasm improvement for preharvest sprouting resistance in Chinese white-grained wheat: An overview of the current strategy. Euphytica 126, 35–38. 10.1023/A:1019679924173

[B53] Yu M. Chen G. Y. Zhang L. Q. Liu Y. X. Liu D. C. Wang J. R. (2014). QTL Mapping for important agronomic traits in synthetic hexaploid wheat derived from *Aegiliops tauschii* ssp. tauschii. J. Integr. Agric. 13, 1835–1844. 10.1016/S2095-3119(13)60655-3

