# Supplementary material for: An Advanced Backcross Population through Synthetic Octaploid Wheat as a “Bridge”: Development and QTL Detection for Seed Dormancy
Source: Front Plant Sci. 2017 Dec 13;8:2123. doi: 10.3389/fpls.2017.02123 (PMC5733556; doi:10.3389/fpls.2017.02123)
Supplement: Supplementary file 1 [file DataSheet1.docx]

**Supplementary Material**

**An advanced backcross population through synthetic octaploid wheat as a ‘bridge’: development and QTL detection for seed dormancy**

**Dale Zhang^1,2+^, Jie He^1+^, Luyu Huang^1^, Cancan Zhang^1^, Yun Zhou^1,2^, Yarui Su^1^, Suoping Li^1,2*^**

**Table S1** Number of introgressed segments in the advanced backcross population.

| Lines | Homozygous segments | Heterozygous segments | Total |
| --- | --- | --- | --- |
| 150196 | 2 | 0 | 2 |
| 150198 | 16 | 1 | 17 |
| 150207 | 26 | 0 | 26 |
| 150212 | 23 | 1 | 24 |
| 150213 | 5 | 2 | 7 |
| 150215 | 0 | 0 | 0 |
| 150217 | 1 | 0 | 1 |
| 150223 | 0 | 0 | 0 |
| 150225 | 0 | 0 | 0 |
| 150226 | 1 | 0 | 1 |
| 150228 | 10 | 0 | 10 |
| 150229 | 2 | 0 | 2 |
| 150230 | 0 | 0 | 0 |
| 150232 | 1 | 0 | 1 |
| 150234 | 9 | 0 | 9 |
| 150238 | 10 | 2 | 12 |
| 150239 | 14 | 0 | 14 |
| 150240 | 25 | 4 | 29 |
| 150257 | 18 | 2 | 20 |
| 150258 | 0 | 0 | 0 |
| 150259 | 12 | 3 | 15 |
| 150260 | 40 | 0 | 40 |
| 150262 | 1 | 0 | 1 |
| 150264 | 7 | 4 | 11 |
| 150265 | 2 | 0 | 2 |
| 150266 | 0 | 0 | 0 |
| 150267 | 0 | 0 | 0 |
| 150270 | 3 | 0 | 3 |
| 150275 | 15 | 3 | 18 |
| 150279 | 3 | 1 | 4 |
| 150284 | 0 | 0 | 0 |
| 150288 | 10 | 3 | 13 |
| 150290 | 13 | 7 | 20 |
| 150300 | 2 | 3 | 5 |
| 150311 | 2 | 1 | 3 |
| 150320 | 0 | 0 | 0 |
| 150329 | 0 | 0 | 0 |
| 150331 | 0 | 0 | 0 |
| 150333 | 0 | 0 | 0 |
| 150345 | 0 | 0 | 0 |
| 150348 | 6 | 3 | 9 |
| 150350 | 0 | 0 | 0 |
| 150357 | 4 | 0 | 4 |
| 150361 | 16 | 0 | 16 |
| 150365 | 1 | 0 | 1 |
| 150368 | 16 | 2 | 18 |
| 150372 | 19 | 1 | 20 |
| 150376 | 0 | 0 | 0 |
| 150379 | 2 | 0 | 2 |
| 150382 | 0 | 0 | 0 |
| 150389 | 0 | 0 | 0 |
| 150394 | 1 | 0 | 1 |
| 150397 | 32 | 0 | 32 |
| 150401 | 1 | 0 | 1 |
| 150407 | 1 | 1 | 2 |
| 150409 | 38 | 4 | 42 |
| 150410 | 2 | 3 | 5 |
| 150418 | 1 | 0 | 1 |
| 150420 | 18 | 11 | 29 |
| 150423 | 0 | 0 | 0 |
| 150426 | 0 | 0 | 0 |
| 150443 | 0 | 0 | 0 |
| 150445 | 2 | 0 | 2 |
| 150447 | 9 | 1 | 10 |
| 150449 | 0 | 0 | 0 |
| 150450 | 0 | 0 | 0 |
| 150457 | 0 | 0 | 0 |
| 150458 | 3 | 0 | 3 |
| 150461 | 5 | 0 | 5 |
| 150467 | 23 | 0 | 23 |
| 150484 | 4 | 0 | 4 |
| 150494 | 28 | 0 | 28 |
| 150500 | 1 | 0 | 1 |
| 152307 | 0 | 0 | 0 |
| 152331 | 0 | 0 | 0 |
| 152370 | 1 | 0 | 1 |
| 152388 | 7 | 0 | 7 |
| 152412 | 11 | 0 | 11 |
| 152417 | 11 | 0 | 11 |
| 152423 | 10 | 1 | 11 |
| 152447 | 0 | 0 | 0 |
| 152459 | 19 | 1 | 20 |
| 152460 | 23 | 1 | 24 |
| 152465 | 1 | 0 | 1 |
| 152486 | 0 | 0 | 0 |
| 152507 | 0 | 0 | 0 |
| 152531 | 20 | 3 | 23 |
| 152543 | 29 | 0 | 29 |
| 152558 | 8 | 2 | 10 |
| 152585 | 0 | 0 | 0 |
| 152615 | 8 | 1 | 9 |
| 152627 | 11 | 2 | 13 |
| 152642 | 18 | 1 | 19 |
| 152663 | 11 | 0 | 11 |
| 152672 | 0 | 0 | 0 |
| 152699 | 2 | 0 | 2 |
| 152720 | 0 | 0 | 0 |
| 152128 | 14 | 4 | 18 |
| 152138 | 20 | 6 | 26 |
| 152143 | 12 | 3 | 15 |
| 152144 | 4 | 0 | 4 |
| 152145 | 1 | 0 | 1 |
| 152149 | 7 | 2 | 9 |
| 151121 | 1 | 0 | 1 |
| 151143 | 2 | 0 | 2 |
| 151144 | 1 | 0 | 1 |
| 151146 | 1 | 0 | 1 |
| 151147 | 11 | 0 | 11 |
| 151157 | 2 | 0 | 2 |
| 151167 | 2 | 0 | 2 |
| 151173 | 0 | 0 | 0 |
| 151190 | 9 | 0 | 9 |
| 151201 | 4 | 6 | 10 |
| 151202 | 9 | 5 | 14 |
| 151207 | 7 | 0 | 7 |
| 151216 | 4 | 0 | 4 |
| 151222 | 10 | 1 | 11 |
| 151224 | 1 | 0 | 1 |
| 151228 | 8 | 2 | 10 |
| 151231 | 8 | 0 | 8 |
| 151233 | 20 | 2 | 22 |
| 151240 | 0 | 0 | 0 |
| 151248 | 1 | 0 | 1 |
| 151251 | 8 | 3 | 11 |
| 151256 | 13 | 0 | 13 |
| 151260 | 1 | 0 | 1 |
| 151267 | 8 | 1 | 9 |
| 151268 | 2 | 0 | 2 |
| 151269 | 12 | 0 | 12 |
| 151272 | 5 | 0 | 5 |
| 151274 | 2 | 0 | 2 |
| 151282 | 0 | 0 | 0 |
| 151289 | 0 | 0 | 0 |
| 151290 | 0 | 0 | 0 |
| 151300 | 15 | 0 | 15 |
| 151301 | 0 | 0 | 0 |
| 151302 | 0 | 0 | 0 |
| 151306 | 1 | 0 | 1 |
| 151308 | 13 | 1 | 14 |
| 151309 | 13 | 3 | 16 |
| 151310 | 22 | 0 | 22 |
| 151321 | 25 | 0 | 25 |
| 151324 | 2 | 2 | 4 |
| 151325 | 1 | 0 | 1 |
| 151327 | 4 | 0 | 4 |
| 151335 | 29 | 1 | 30 |
| 151337 | 2 | 3 | 5 |
| 151344 | 25 | 0 | 25 |
| 151346 | 1 | 0 | 1 |
| 151357 | 16 | 6 | 22 |
| 151360 | 1 | 0 | 1 |
| 151361 | 0 | 1 | 1 |
| 151365 | 2 | 0 | 2 |
| 151369 | 1 | 0 | 1 |
| 151379 | 1 | 0 | 1 |
| 151390 | 6 | 3 | 9 |
| 151394 | 1 | 0 | 1 |
| 151397 | 0 | 1 | 1 |
| 151414 | 13 | 1 | 14 |
| 151416 | 8 | 1 | 9 |
| 151423 | 4 | 0 | 4 |
| 151424 | 3 | 1 | 4 |
| 151426 | 3 | 1 | 4 |
| 151436 | 4 | 1 | 5 |
| 151442 | 7 | 0 | 7 |
| 151446 | 2 | 1 | 3 |
| 151448 | 17 | 0 | 17 |
| 151459 | 7 | 1 | 8 |
| 151464 | 3 | 0 | 3 |
| 151468 | 3 | 0 | 3 |
| 151469 | 2 | 0 | 2 |
| 151470 | 7 | 0 | 7 |
| 151471 | 9 | 1 | 10 |
| 151473 | 24 | 0 | 24 |
| 151477 | 0 | 0 | 0 |
| 151478 | 1 | 0 | 1 |
| 151481 | 2 | 0 | 2 |
| 151488 | 4 | 0 | 4 |
| 151490 | 1 | 3 | 4 |
| 151493 | 13 | 3 | 16 |
| 151495 | 9 | 1 | 10 |
| 151498 | 10 | 2 | 12 |
| 151506 | 6 | 3 | 9 |
| 151515 | 9 | 1 | 10 |
| 151517 | 22 | 0 | 22 |
| 151520 | 14 | 0 | 14 |
| 151529 | 24 | 2 | 26 |
| 151541 | 11 | 2 | 13 |
| 151559 | 2 | 0 | 2 |
| 151284 | 1 | 0 | 1 |
| 150444 | 1 | 0 | 1 |
| 151247 | 2 | 0 | 2 |
| 150497 | 7 | 0 | 7 |
| 150502 | 2 | 0 | 2 |
| 143314 | 2 | 0 | 2 |
| 152888 | 7 | 0 | 7 |
| 153296 | 1 | 0 | 1 |
| 153416 | 1 | 0 | 1 |
| 153440 | 1 | 0 | 1 |
| 153461 | 1 | 0 | 1 |
| 153470 | 6 | 3 | 9 |
| Total | 1363 | 159 | 1522 |

**Table S2** The seed dormancy rate (Dor) of the advanced backcross population (201 lines) on the day 3, 5 and 7.

|  | Seed dormancy rate (%) | | |
| --- | --- | --- | --- |
| Lines | 3 day | 5 day | 7 day |
| 150196 | 9.00 | 8.00 | 8.00 |
| 150198 | 54.00 | 53.00 | 51.00 |
| 150207 | 34.00 | 26.00 | 26.00 |
| 150212 | 98.00 | 96.00 | 96.00 |
| 150213 | 72.00 | 50.00 | 35.00 |
| 150215 | 71.00 | 61.00 | 60.00 |
| 150217 | 52.00 | 50.00 | 48.00 |
| 150223 | 81.00 | 80.00 | 74.00 |
| 150225 | 55.00 | 54.00 | 53.00 |
| 150226 | 63.00 | 60.00 | 57.00 |
| 150228 | 62.00 | 62.00 | 62.00 |
| 150229 | 51.00 | 43.00 | 40.00 |
| 150230 | 65.00 | 63.00 | 63.00 |
| 150232 | 75.00 | 74.00 | 74.00 |
| 150234 | 13.00 | 8.00 | 8.00 |
| 150238 | 93.00 | 93.00 | 90.00 |
| 150239 | 57.00 | 54.00 | 51.00 |
| 150240 | 96.00 | 94.00 | 92.00 |
| 150257 | 21.00 | 3.00 | 2.00 |
| 150258 | 0.00 | 0.00 | 0.00 |
| 150259 | 94.00 | 94.00 | 94.00 |
| 150260 | 88.00 | 86.00 | 76.00 |
| 150262 | 5.00 | 0.00 | 0.00 |
| 150264 | 72.00 | 60.00 | 60.00 |
| 150265 | 0.00 | 0.00 | 0.00 |
| 150266 | 5.00 | 5.00 | 5.00 |
| 150267 | 0.00 | 0.00 | 0.00 |
| 150270 | 72.00 | 71.00 | 67.00 |
| 150275 | 5.00 | 4.00 | 2.00 |
| 150279 | 2.00 | 0.00 | 0.00 |
| 150284 | 0.00 | 0.00 | 0.00 |
| 150288 | 13.00 | 9.00 | 9.00 |
| 150290 | 14.00 | 8.00 | 8.00 |
| 150300 | 90.00 | 86.00 | 84.00 |
| 150311 | 48.00 | 40.00 | 40.00 |
| 150320 | 91.00 | 90.00 | 89.00 |
| 150329 | 0.00 | 0.00 | 0.00 |
| 150331 | 3.00 | 2.00 | 2.00 |
| 150333 | 53.00 | 51.00 | 44.00 |
| 150345 | 44.00 | 41.00 | 38.00 |
| 150348 | 7.00 | 0.00 | 0.00 |
| 150350 | 27.00 | 24.00 | 21.00 |
| 150357 | 2.00 | 2.00 | 2.00 |
| 150361 | 57.00 | 56.00 | 56.00 |
| 150365 | 17.00 | 16.00 | 14.00 |
| 150368 | 43.00 | 29.00 | 20.00 |
| 150372 | 100.00 | 100.00 | 96.00 |
| 150376 | 25.00 | 13.00 | 12.00 |
| 150379 | 0.00 | 0.00 | 0.00 |
| 150382 | 8.00 | 0.00 | 0.00 |
| 150389 | 0.00 | 0.00 | 0.00 |
| 150394 | 69.00 | 69.00 | 69.00 |
| 150397 | 51.00 | 51.00 | 44.00 |
| 150401 | 49.00 | 48.00 | 47.00 |
| 150407 | 43.00 | 43.00 | 43.00 |
| 150409 | 100.00 | 100.00 | 98.00 |
| 150410 | 6.00 | 4.00 | 4.00 |
| 150418 | 29.00 | 25.00 | 20.00 |
| 150420 | 82.00 | 78.00 | 70.00 |
| 150423 | 7.00 | 3.00 | 2.00 |
| 150426 | 15.00 | 9.00 | 4.00 |
| 150443 | 8.00 | 8.00 | 8.00 |
| 150445 | 4.00 | 2.00 | 0.00 |
| 150447 | 0.00 | 0.00 | 0.00 |
| 150449 | 3.00 | 3.00 | 3.00 |
| 150450 | 41.00 | 41.00 | 33.00 |
| 150457 | 6.00 | 2.00 | 2.00 |
| 150458 | 27.00 | 26.00 | 26.00 |
| 150461 | 0.00 | 0.00 | 0.00 |
| 150467 | 70.00 | 70.00 | 70.00 |
| 150484 | 83.00 | 81.00 | 78.00 |
| 150494 | 76.00 | 70.00 | 70.00 |
| 150500 | 4.00 | 3.00 | 3.00 |
| 152307 | 59.00 | 52.00 | 51.00 |
| 152331 | 16.00 | 16.00 | 16.00 |
| 152370 | 4.00 | 2.00 | 2.00 |
| 152388 | 15.00 | 11.00 | 10.00 |
| 152412 | 61.00 | 61.00 | 61.00 |
| 152417 | 0.00 | 0.00 | 0.00 |
| 152423 | 25.00 | 23.00 | 22.00 |
| 152447 | 0.00 | 0.00 | 0.00 |
| 152459 | 12.00 | 9.00 | 8.00 |
| 152460 | 0.00 | 0.00 | 0.00 |
| 152465 | 45.00 | 39.00 | 37.00 |
| 152486 | 0.00 | 0.00 | 0.00 |
| 152507 | 7.00 | 2.00 | 2.00 |
| 152531 | 6.00 | 6.00 | 6.00 |
| 152543 | 96.00 | 84.00 | 82.00 |
| 152558 | 2.00 | 2.00 | 2.00 |
| 152585 | 29.00 | 11.00 | 10.00 |
| 152615 | 0.00 | 0.00 | 0.00 |
| 152627 | 55.00 | 50.00 | 50.00 |
| 152642 | 53.00 | 49.00 | 42.00 |
| 152663 | 44.00 | 40.00 | 40.00 |
| 152672 | 5.00 | 0.00 | 0.00 |
| 152699 | 61.00 | 59.00 | 59.00 |
| 152720 | 47.00 | 45.00 | 40.00 |
| 152128 | 54.00 | 28.00 | 20.00 |
| 152138 | 100.00 | 100.00 | 98.00 |
| 152143 | 98.00 | 90.00 | 86.00 |
| 152144 | -100.00 | -100.00 | -100.00 |
| 152145 | 13.00 | 12.00 | 11.00 |
| 152149 | 39.00 | 36.00 | 31.00 |
| 151121 | 61.00 | 59.00 | 54.00 |
| 151143 | 90.00 | 90.00 | 88.00 |
| 151144 | 7.00 | 2.00 | 2.00 |
| 151146 | 4.00 | 4.00 | 3.00 |
| 151147 | 0.00 | 0.00 | 0.00 |
| 151157 | 0.00 | 0.00 | 0.00 |
| 151167 | 77.00 | 67.00 | 64.00 |
| 151173 | 9.00 | 8.00 | 8.00 |
| 151190 | 47.00 | 45.00 | 40.00 |
| 151201 | 13.00 | 9.00 | 7.00 |
| 151202 | 2.00 | 0.00 | 0.00 |
| 151207 | 0.00 | 0.00 | 0.00 |
| 151216 | 7.00 | 4.00 | 3.00 |
| 151222 | -100.00 | -100.00 | -100.00 |
| 151224 | 0.00 | 0.00 | 0.00 |
| 151228 | 0.00 | 0.00 | 0.00 |
| 151231 | 0.00 | 0.00 | 0.00 |
| 151233 | 96.00 | 88.00 | 80.00 |
| 151240 | 0.00 | 0.00 | 0.00 |
| 151248 | -100.00 | -100.00 | -100.00 |
| 151251 | 15.00 | 7.00 | 2.00 |
| 151256 | 41.00 | 37.00 | 31.00 |
| 151260 | 0.00 | 0.00 | 0.00 |
| 151267 | 25.00 | 24.00 | 4.00 |
| 151268 | 55.00 | 53.00 | 48.00 |
| 151269 | 53.00 | 47.00 | 45.00 |
| 151272 | 0.00 | 0.00 | 0.00 |
| 151274 | 0.00 | 0.00 | 0.00 |
| 151282 | 87.00 | 87.00 | 86.00 |
| 151289 | 0.00 | 0.00 | 0.00 |
| 151290 | 4.00 | 0.00 | 0.00 |
| 151300 | 0.00 | 0.00 | 0.00 |
| 151301 | 2.00 | 2.00 | 0.00 |
| 151302 | 13.00 | 9.00 | 8.00 |
| 151306 | 17.00 | 13.00 | 11.00 |
| 151308 | 0.00 | 0.00 | 0.00 |
| 151309 | 54.00 | 24.00 | 24.00 |
| 151310 | 78.00 | 74.00 | 66.00 |
| 151321 | 76.00 | 56.00 | 32.00 |
| 151324 | 53.00 | 31.00 | 30.00 |
| 151325 | 25.00 | 20.00 | 19.00 |
| 151327 | 15.00 | 12.00 | 10.00 |
| 151335 | 2.00 | 2.00 | 0.00 |
| 151337 | -100.00 | -100.00 | -100.00 |
| 151344 | 98.00 | 92.00 | 90.00 |
| 151346 | 2.00 | 0.00 | 0.00 |
| 151357 | 18.00 | 2.00 | 0.00 |
| 151360 | 47.00 | 41.00 | 36.00 |
| 151361 | 9.00 | 9.00 | 9.00 |
| 151365 | 0.00 | 0.00 | 0.00 |
| 151369 | 8.00 | 4.00 | 4.00 |
| 151379 | 87.00 | 82.00 | 80.00 |
| 151390 | 15.00 | 13.00 | 10.00 |
| 151394 | 98.00 | 98.00 | 94.00 |
| 151397 | -100.00 | -100.00 | -100.00 |
| 151414 | 71.00 | 67.00 | 64.00 |
| 151416 | 71.00 | 27.00 | 27.00 |
| 151423 | 62.00 | 49.00 | 47.00 |
| 151424 | 20.00 | 16.00 | 12.00 |
| 151426 | 32.00 | 15.00 | 15.00 |
| 151436 | 51.00 | 47.00 | 24.00 |
| 151442 | 67.00 | 55.00 | 51.00 |
| 151446 | 45.00 | 37.00 | 14.00 |
| 151448 | 100.00 | 98.00 | 90.00 |
| 151459 | 10.00 | 2.00 | 0.00 |
| 151464 | 7.00 | 0.00 | 0.00 |
| 151468 | 0.00 | 0.00 | 0.00 |
| 151469 | 0.00 | 0.00 | 0.00 |
| 151470 | 5.00 | 4.00 | 3.00 |
| 151471 | 2.00 | 2.00 | 2.00 |
| 151473 | 5.00 | 2.00 | 1.00 |
| 151477 | 31.00 | 9.00 | 6.00 |
| 151478 | 0.00 | 0.00 | 0.00 |
| 151481 | 2.00 | 2.00 | 2.00 |
| 151488 | 59.00 | 43.00 | 43.00 |
| 151490 | 96.00 | 96.00 | 94.00 |
| 151493 | 54.00 | 44.00 | 44.00 |
| 151495 | 98.00 | 96.00 | 95.00 |
| 151498 | 98.00 | 96.00 | 94.00 |
| 151506 | -100.00 | -100.00 | -100.00 |
| 151515 | 0.00 | 0.00 | 0.00 |
| 151517 | 98.00 | 96.00 | 94.00 |
| 151520 | 51.00 | 51.00 | 39.00 |
| 151529 | 98.00 | 98.00 | 88.00 |
| 151541 | 0.00 | 0.00 | 0.00 |
| 151559 | 5.00 | 4.00 | 3.00 |
| 151284 | 5.00 | 5.00 | 3.00 |
| 150444 | 13.00 | 9.00 | 9.00 |
| 151247 | 9.00 | 7.00 | 6.00 |
| 150497 | 2.00 | 2.00 | 2.00 |
| 150502 | 13.00 | 8.00 | 7.00 |
| 143314 | 75.00 | 73.00 | 73.00 |
| 152888 | 27.00 | 4.00 | 3.00 |
| 153296 | 5.00 | 5.00 | 5.00 |
| 153416 | 4.00 | 2.00 | 2.00 |
| 153440 | 79.00 | 79.00 | 75.00 |
| 153461 | 21.00 | 15.00 | 13.00 |
| 153470 | 92.00 | 84.00 | 80.00 |

Note: “-100.00” indicates missing data.

**Table S3** Correlation coefficients between 2 replications associated with Dor in the advanced backcross population.

| Traits | Correlation coefficient |
| --- | --- |
| Dor 3-R1/Dor 3-R2 | 0.969^**^ |
| Dor 5- R1/Dor 5- R2 | 0.981^**^ |
| Dor 7- R1/Dor 7- R2 | 0.982^**^ |

**: correlation is significant at the 0.01 level (2-tailed).

Dor 3-R1/Dor 3-R2: 2 replications of seed dormancy rate on the day 3, Dor 5-R1/Dor 5-R2: 2 replications of seed dormancy rate on the day 5, Dor 7-R1/Dor 7-R2: 2 replications of seed dormancy rate on the day 7.

**
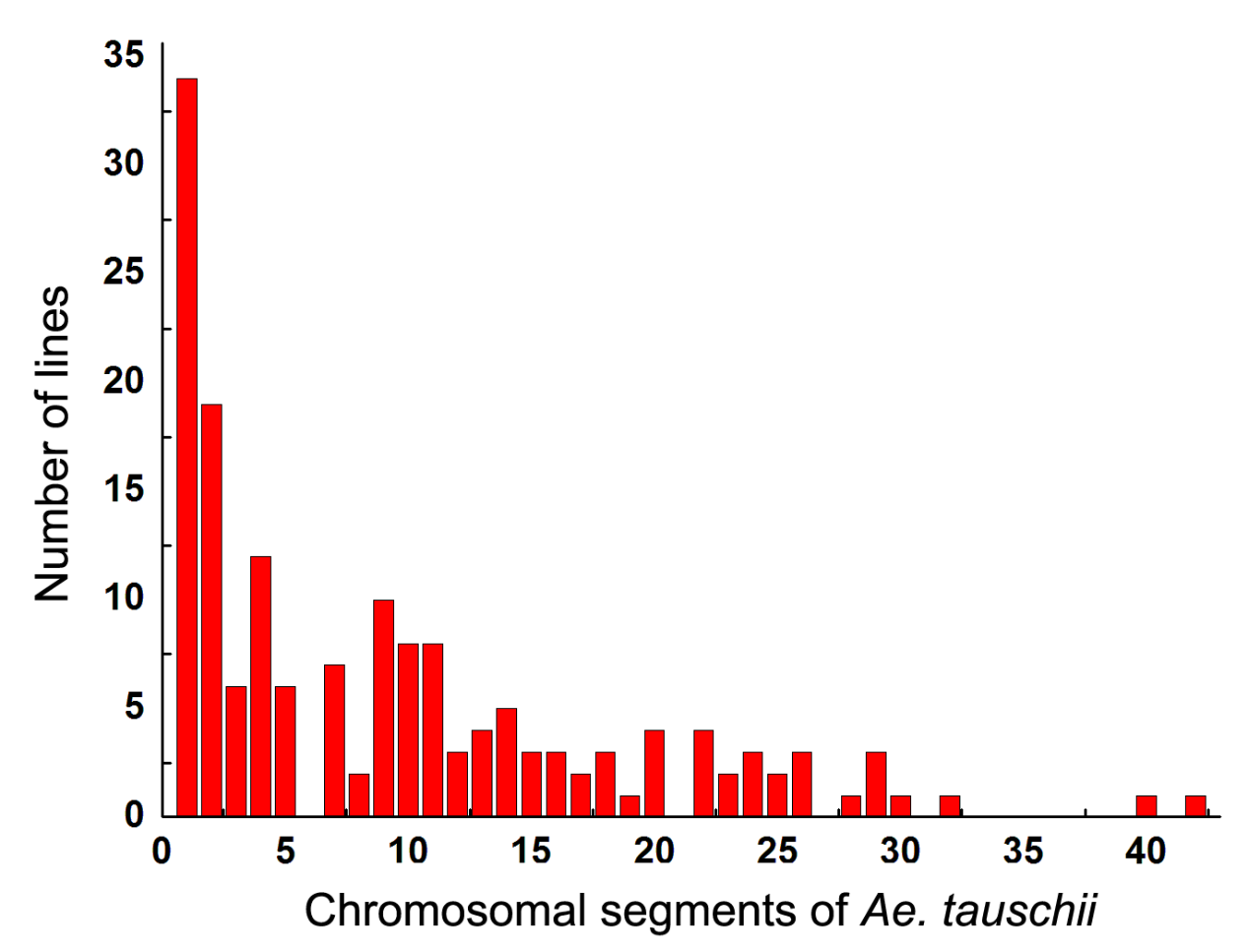
**

**Fig.S1** Distribution of chromosome segments from *Ae. tauschii* accession T093 among the 201 lines.


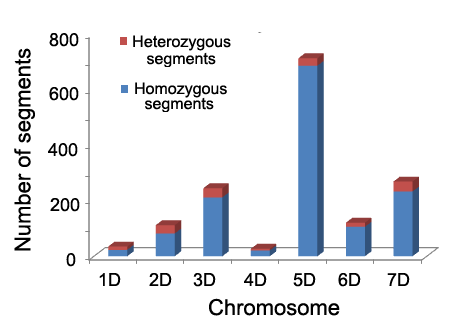


**Fig.S2** Distribution of chromosome segments from *Ae. tauschii* accession T093 in the wheat D genome.
